# Integrated analysis of RNA-seq in hepatocellular carcinoma reveals competing endogenous RNA network composed of circRNA, lncRNA, and mRNA

**DOI:** 10.1097/MD.0000000000032915

**Published:** 2023-02-22

**Authors:** Fuyin Zhou, Qingsong Kang, Junbo Ma, Jie Cai, Ying Chen, Kai Qu, Feibo Li

**Affiliations:** a Department of General Surgery, People’s Hospital of Putuo District, Zhoushan, Zhejiang, China.

**Keywords:** ceRNA, circRNA, HCC, lncRNA

## Abstract

**Background:**

Circular RNAs (circRNAs) and long non-coding RNAs (lncRNAs) have been hypothesized to have important roles in the etiology of hepatocellular carcinoma (HCC). However, the synergistic effect of circRNA and lncRNA in the pathogenesis of HCC has rarely been studied.

**Methods:**

In this study, the Gene Expression Omnibus database was used to get the expression profiles of circRNAs, micro RNAs (miRNAs), lncRNAs, and messenger RNAs (mRNAs) in HCC tissues and normal tissues. The accession numbers for this database are GSE101728, GSE155949, and GSE108724. We found 291 differentially overexpressed lncRNAs and 541 differentially overexpressed mRNA in GSE101728, 30 differentially overexpressed circRNA in GSE155949, and 48 significantly downregulated miRNA in GSE198724. Meanwhile, based on Pearson correlation test, we established lncRNA–mRNA networks. We constructed lncRNA/circRNA–miRNA pairs through Starbase database prediction and identified the common miRNAs. The intersection of co-predicted miRNAs and the 48 significantly low expression miRNAs in GSE198724 were included in the following study. miRDB, Targetscan, miRwalk, and lncRNA-related mRNA jointly determined the miRNA–mRNA portion of the circRNA/lncRNA–miRNA–mRNA co-expression network. And, among 55 differentially expressed mRNA in circRNA/lncRNA–miRNA–mRNA network, CPEB3, EFNB3, FATA4, growth hormone receptor, GSTZ1, KLF8, MFAP4, PAIP2B, PHACTR3, PITPNM3, RPS6KA6, RSPO3, SLITRK6, SMOC1, STEAP4, SYT1, TMEM132E, TSPAN11, and ZFPM2 were intimately related to the prognosis of HCC patients in Kaplan–Meier plotter analysis (*P* < .05).

**Conclusion:**

We have discovered that the prognosis-related lncRNAs/circRNAs–miRNA–mRNA network plays a significant role in the pathogenesis of HCC. These findings may offer fresh perspectives for further research into the pathogenesis of HCC and the search for novel treatments for HCC.

## 1. Introduction

Approximately 900,000 new cases of liver cancer are diagnosed each year and account for >830,000 deaths worldwide in 2020.^[1]^ Ninety percent of liver cancer is hepatocellular carcinoma (HCC). Nearly 90% of HCC cases are caused by aflatoxin exposure, alcohol usage, and chronic viral hepatitis (B and C).^[2]^ Patients’ recurrence-free survival and overall survival (OS) are still relatively short despite major improvements in HCC detection and therapy.^[3]^ Current therapies have a 5-year survival rate of roughly 20% because of factors like poor surgical prognosis, advanced-stage diagnosis, restricted chemoembolization, and multikinase inhibitors (sorafenib, which is only helpful for 30% of patients and causes drug resistance to develop within 6 months).^[4]^ This demonstrates the significance of elucidating the mechanisms behind HCC development and identifying effective molecular biomarkers.

Less than 3% of the human genome codes for exons, but over 97% of the genome is translated into non-coding RNAs (ncRNAs), such as microRNAs and long non-coding RNAs (lncRNAs). This information was recently discovered through breakthroughs in transcriptome sequencing. ncRNAs are non-protein-coding RNA molecules that play key roles in DNA replication, RNA splicing, translation, and epigenetic regulation.^[5]^ The competitive endogenous RNA (ceRNA) theory, first proposed by Salmena et al, is largely accepted by the scientific community.^[6]^ lncRNAs can function in the ceRNA network as endogenous molecular sponges of miRNAs and indirectly control the production of messenger RNAs. This makes it possible to relate the role of ncRNAs to that of mRNAs, which code for proteins.^[7]^ Both lncRNA and circular RNAs (circRNA) can influence the expression of mRNA of target gene through the ceRNA network, but there aren’t many studies indicating that these 2 RNAs may regulate mRNA expression by competing for binding to the same miRNA.

It is still unclear how circRNA and lncRNA affect HCC. A thorough analysis of lncRNA and circRNA is urgently needed to investigate the function of the lncRNA/circRNA–miRNA–mRNA ceRNA network in HCC. In the present study, the Gene Expression Omnibus database is used to identify differentially expressed (DE) mRNA, DEmiRNA, DElncRNA, and DEcircRNA between HCC and normal tissues. The target miRNAs of DEcircRNAs and DElncRNAs were predicted separately and crossed to produce common miRNAs. There have been identified lncRNA–miRNA–mRNA and circRNA–miRNA–mRNA ceRNA networks for HCC. However, only a few studies have examined lncRNAs and circRNAs together in the ceRNA network of HCC. In this study, we developed the circRNA/lncRNA–miRNA–mRNA ceRNA network based on expression correlation analysis and database prediction. The prognosis of patients is greatly impacted by DEmRNA in the ceRNA network. Consequently, our findings may reveal novel HCC etiologies and provide innovative therapeutic strategies.

## 2. Methods

### 2.1. Public microarray datasets preparation

Three microarray datasets including ncRNA, GSE101728,^[8]^ GSE155949,^[9]^ and GSE108724,^[8]^ were found by searching the National Center for Biotechnology Information Gene Expression Omnibus database repository with the keywords, RNA and hepatocellular carcinoma. The lncRNA and mRNA microarray GSE101728 (GPL21047 Agilent-074348 Human LncRNA v6 4X180K) containing 7 human HCC tissue and 7 tumor adjacent-tissues lncRNA and mRNA expression data. miRNA microarray GSE108724 (GPL20712 Agilent 070156 Human miRNA) containing 7 human HCC tissues and 7 tumor-adjacent tissues miRNA expression data. circRNA microarray GSE155949 (GPL21825 074301 Arraystar Human CircRNA microarray V2) containing 49 human HCC tissues and 49 tumor-adjacent tissues circRNA expression data.

### 2.2. Identification of DERNAs

The normalize Between Arrays function of the “limma” R package was used to normalize the transcriptome data. The DEmRNAs, DElncRNAs in HCC, and normal samples were identified using the same R program with log2FC > 2 as the threshold for statistical significance and *P* value of .05 as the cutoff. The DEmiRNAs were identified with log2FC < −1 and *P* < .05 as the threshold for statistical significance. DEcircRNAs were identified with log2FC > 1 and *P* < .05 as the threshold for statistical significance. The DERNAs’ heatmap and volcanos were created using the R packages “pheatmap” and “ggplot2.” DERNA intersections were calculated using a Venn diagram.

### 2.3. Establishment of DElncRNA–DEmRNA

The lncRNA–mRNA coexpression network shows how differently expressed mRNAs and lncRNAs interact. The normalized signal intensities of DEmRNA and DElncRNA expression levels provide the foundation of this construct. Pearson correlations were utilized to calculate statistically significant relationships in the DElncRNA–DEmRNA co-expression network described here. We believe that DElncRNA–DEmRNA pairs conforming to *ρ* > 0.8 and *P* < .05 are closely correlated.

### 2.4. DEcircRNA/lncRNA–miRNA

The regulatory interaction between miRNA and lncRNA was investigated using Starbase (http://starbase.sysu.edu.cn/),^[10]^ and the lncRNA–miRNA ceRNA network was built using Cytoscape software.

### 2.5. Construction of lncRNA/circRNA–miRNA–mRNA network

Based on the targeting interactions, a circRNA/lncRNA–miRNA–mRNA ceRNA network was constructed. The intersection of expected miRNA in both lncRNA–miRNA and circRNA–miRNA pairings formed the miRNAs in circRNA/lncRNA–miRNA–mRNA coexpression network. The TargetScan, miRDB, and miRwalk2.0 databases were used to find the target mRNAs of these miRNAs.^[11–13]^ We only chose those miRNA–mRNA interaction pairings that overlap in all 3 databases for additional investigation to increase the reliability of the results. And not all of the mRNAs predicted in the 3 data sets are part of our ceRNA network; only mRNAs that are strongly associated with the production of lncRNA and circRNA may be judged as ceRNA network mRNAs.

### 2.6. Kaplan–Meier (KM) plotter and Human Protein Atlas (HPA) database analysis

Using the KM plotter (www.kmplot.com),^[14]^ an online database of gene expression data and clinical data, the predictive relevance of DEmRNA in ceRNA network was assessed. This database contains information on breast cancer, gastric cancer, ovarian cancer, lung cancer, and gastrointestinal cancer. The patient samples were split into 2 cohorts based on the gene’s median expression in order to evaluate the prognostic significance of that gene (high vs low expression). We used a KM survival plot to examine the OS of patients with HCC. Log-rank P, hazard ratios, and 95% confidence intervals were computed. Statistical significance was set at *P* < 0.05.

The protein expression patterns in human cell and tissue samples are available through the HPA online tool (https://www.proteinatlas.org). The HPA online tool was used to confirm the immunohistochemistry protein expression of potential hub genes.^[15]^

## 3. Results

### 3.1. Identification of DElncRNA, DEcircRNA, DEmiRNA, and DEmRNAs between tumor-adjacent and HCC samples

**Figure 1.**
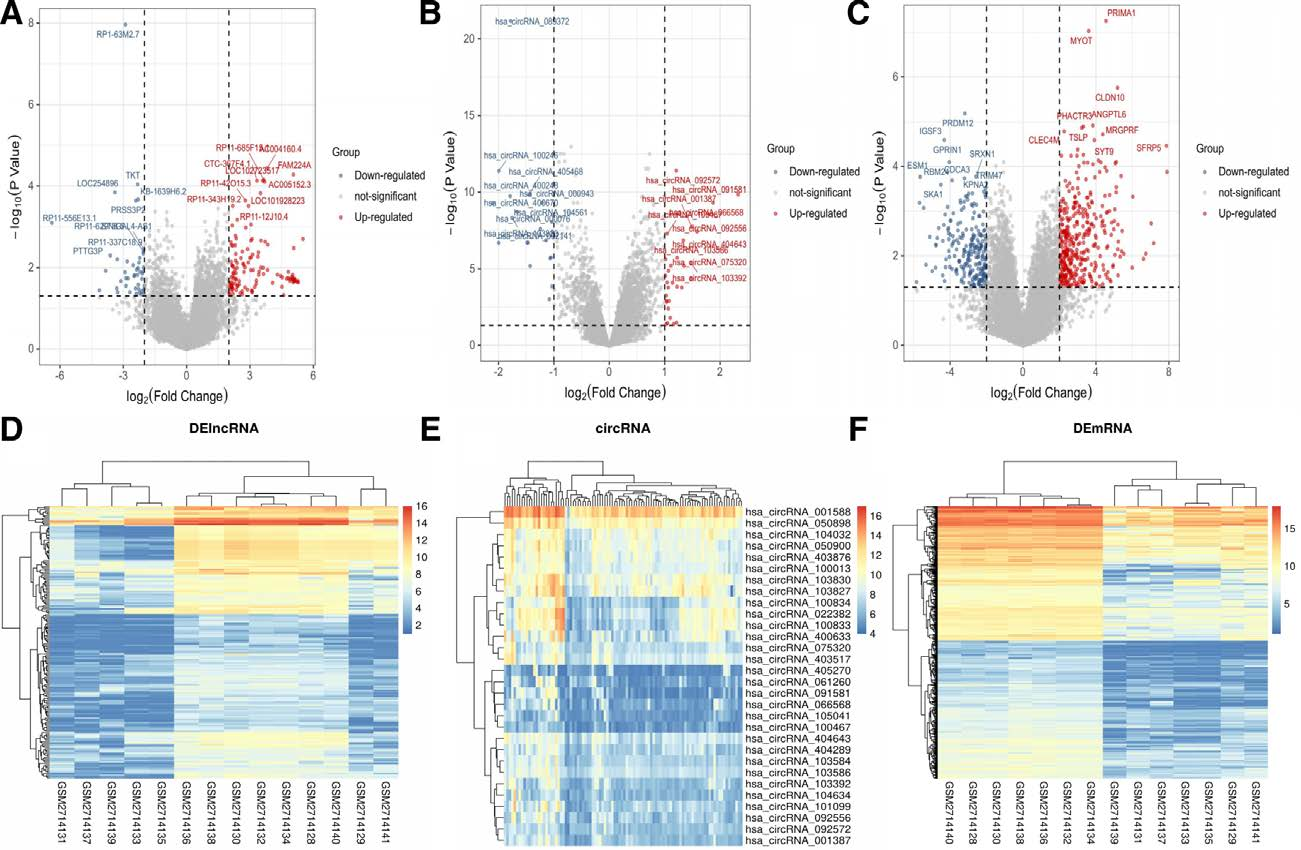
DERNAs between HCC and normal tissues. (A) Differentially expressed lncRNA in GSE101728. The gray part is the molecule with no statistically significant expression difference, the red part is the high expression difference molecule, and the blue part is the low expression difference molecule. (B) DEcircRNA in GSE155949. (C) DEmRNA in GSE101728. (D) Heatmaps of DElncRNA in GSE101728. (E) Heatmaps of DEcircRNA in GSE155949. (F) Heatmaps of DEmRNA in GSE101728. DEcircRNA = differentially expressed circular RNA, DE = differentially expressed, HCC = hepatocellular carcinoma, lncRNA = long non-coding RNA.

**Figure 2.**
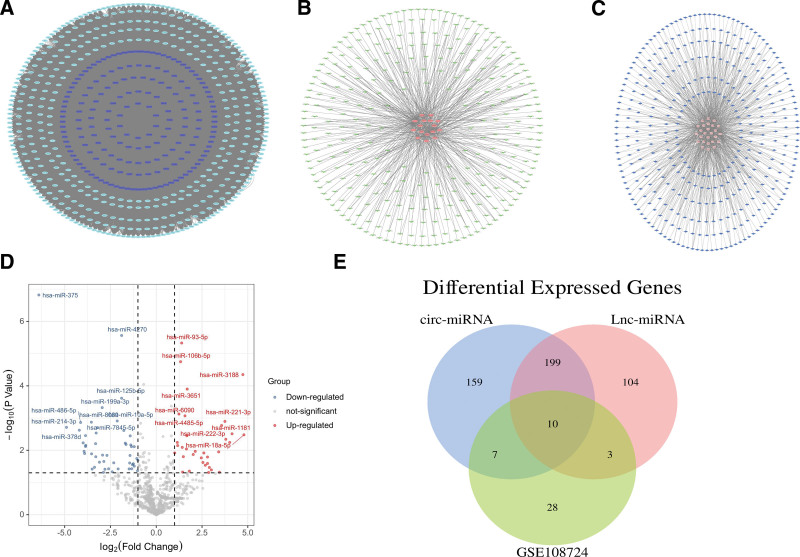
Establishment of miRNA molecules in co-expression networks. (A) lncRNA–mRNA pairs based on Pearson correlation analysis. (B) Predicted lncRNA–miRNA networks. (C) Predicted circRNA–miRNA networks. (D) DEmiRNA in GSE198724. (E) Common miRNAs in three sets. circRNA = circular RNA, DE = differentially expressed, lncRNA = long non-coding RNA, mRNA = messenger RNA.

In GSE101728 datasets, 29809 DEmRNA and 35357 DElncRNA were identified. According to the cutoff FDR < 0.05 and logFC > 2, 291 differentially overexpressed lncRNAs and 541 differentially overexpressed mRNAs were identified using differential gene expression analysis between cancer tissues and normal adjacent tissues (Fig. 1A and C). The GSE155949 datasets included circRNA data, in which 10592 DEcircRNAs were examined when comparing HCC tissues with adjacent tumor tissues (Fig. 1B). Among them, 30 circRNA met the criterion of logFC > 1, FDR < 0.05. For miRNA, 48 significantly downregulated miRNA in GSE198724 were detected (Fig. 2D). Heatmaps of DERNAs in their respective datasets were shown in Figure 1D–F.

### 3.2. DElncRNA–mRNA co-expression network

To find DElncRNA–DEmRNA pairs, Pearson correlation analysis was used on DElncRNAs and DEmRNAs (*P* < .05 and *R* ≥ 0.6) (Fig. 2A). Besides, lncRNA and mRNA with unrelated expression can be excluded using Pearson correlation analysis to establish ceRNA. In general, 188 DElncRNAs and 465 DEmRNA formed a DElncRNA–DEmRNA network composed of 650 nodes and 46,725 edges.

### 3.3. DElnc/circRNA–miRNA

The pairs of DElncRNA/circRNA–miRNA were matched using the Starbase database. Because lncRNA/circRNA and miRNAs are not identical, and some lncRNAs/circRNAs in the database do not predict miRNAs, therefore 16 lncRNAs and 316 miRNAs formed 467 potential lncRNA–miRNA pairs. A total of 916 possible circRNA–miRNA couples were produced by 26 lncRNAs and 375 miRNAs (Fig. 2B–C).

From the GSE198724, a total of 2549 miRNAs were identified, and we found 48 downregulated miRNAs with logFC < −1, *P* < .05 (Fig. 2D). miRNAs that were detected in 48 miRNAs with significantly low expression in the GSE198724 dataset and shared by circRNA–miRNA and lncRNA–miRNA were chosen as common miRNAs in the co-expression network for further study. As we can see in Figure 2E, hsa-miR-214-3p, hsa-let-7b-5p, hsa-let-7c-5p, hsa-miR-195-5p, hsa-miR-424-5p, hsa-miR-142-5p, hsa-miR-145-5p, hsa-miR-378a-3p, hsa-miR-378i and hsa-miR-378d meet all these criteria.

### 3.4. miRNA–mRNA targeting relationship prediction

**Figure 3.**
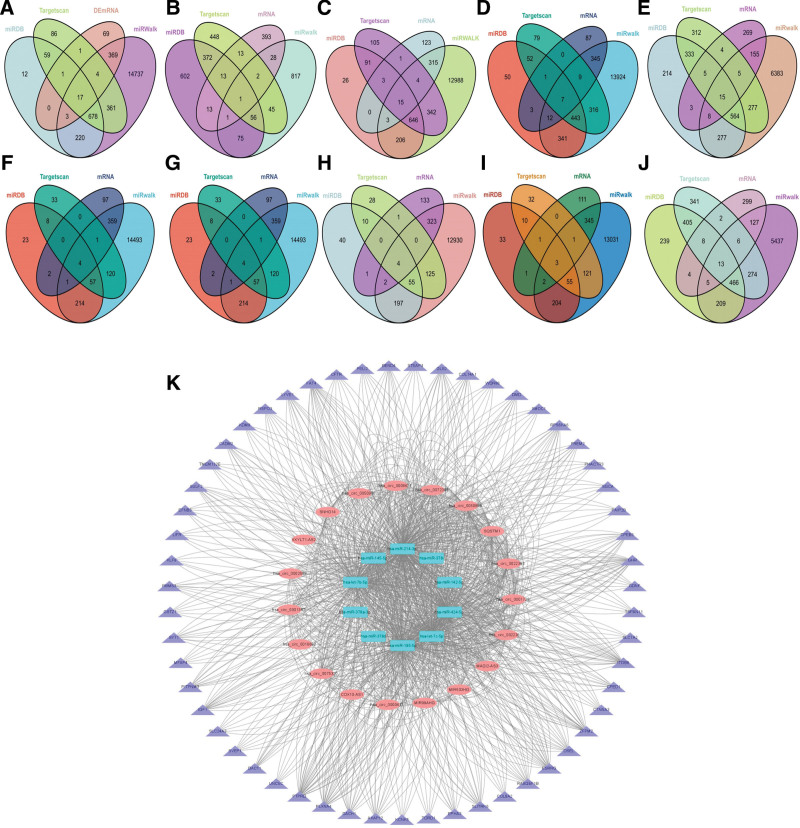
Establishment of miRNA–mRNA and circRNA/lncRNA–miRNA–mRNA networks. (A–I) 10 miRNA that is lowly expressed in HCC tissue and can combine with lncRNA and circRNA. (K) circRNA/lncRNA–miRNA–mRNA co-expression network. circRNA = circular RNA, HCC = hepatocellular carcinoma, lncRNA = long non-coding RNA.

mRNAs targeted by 10 miRNAs were predicted by Targetscan, miRDB, and miRwalk databases. To increase prediction accuracy even more, we took the intersection of each miRNA’s prediction findings from the 3 databases. In addition to this, the 3 databases’ projected miRNA–mRNA combinations are not the final mRNAs that our co-expression network wishes to investigate. This co-expression network contained only the anticipated mRNAs found in DEmRNAs linked to lncRNA expression that we previously screened (Fig. 3A–J).

### 3.5. Establishment of circRNA/lncRNA–miRNA–mRNA ceRNA network in HCC

Based on a ceRNA network mediated by DElncRNAs and DEcircRNAs, a total of 6 DElncRNAs, 13 DEcircRNAs, and 584 miRNA–mRNA pairs were used to construct a ceRNA network co-mediated by DElncRNAs and DEcircRNAs. As shown in the figure below (Fig. 3K), the network involves 6 lncRNAs, 13 circRNAs, 10 miRNAs, and 55 mRNAs. ceRNA network shows that these lncRNAs and circRNAs can not only participate in the regulation of biological processes independently but also jointly affect downstream gene expression and participate in the occurrence and development of HCC as miRNA sponges.

### 3.6. KM plotter analysis of DEmRNA in ceRNA network

**Figure 4.**
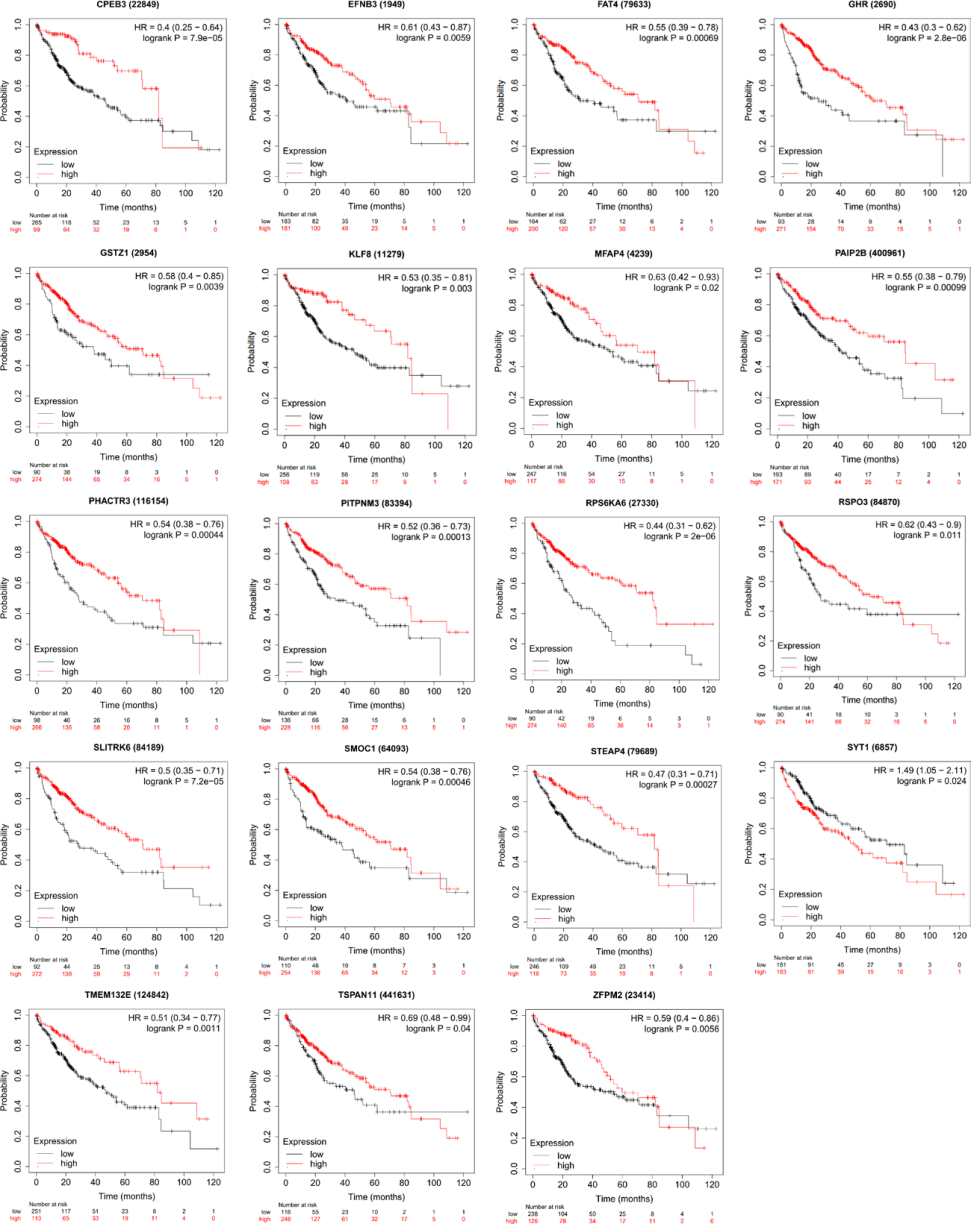
DEmRNA survival analysis in KM plotter database. DE = differentially expressed, KM = Kaplan–Meier.

**Figure 5.**
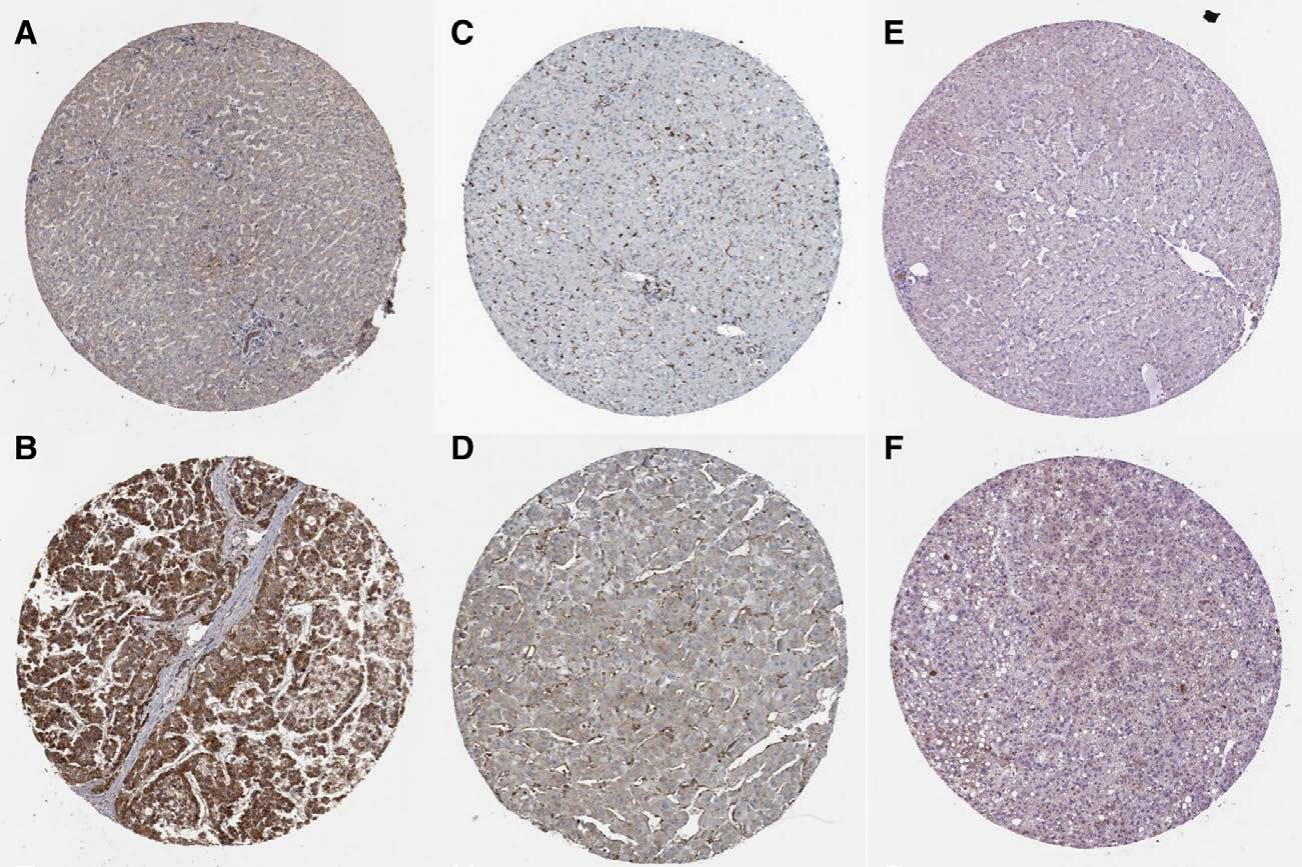
Representative IHC images of DEmRNA in normal liver tissues and HCC tissues in HPA database. (A) RPS6KA6 in normal liver tissues. (B) RPS6KA6 in HCC tissues. (C) RSOP3 in normal liver tissues. (D) RSOP3 in HCC tissues. (E) TMEM132E in normal tissues. (F) TMEM132E in HCC tissues. DE = differentially expressed, HCC = hepatocellular carcinoma, HPA = Human Protein Atlas, IHC = .

By using a KM plotter database, we examined the relationship between the transcription levels of the DEmRNA in the ceRNA network and patient survival. Among 55 DEmRNA, higher expressions of 19 mRNA, namely CPEB3, EFNB3, FATA4, growth hormone receptor (GHR), GSTZ1, KLF8, MFAP4, PAIP2B, PHACTR3, PITPNM3, RPS6KA6, RSPO3, SLITRK6, SMOC1, STEAP4, TMEM132E, TSPAN11, and ZFPM2 were associated with longer OS in 364 HCC patients (*P* < .05) (Fig. 4). Besides, utilizing HPA databases, the expression of DEmRNA at the protein level was further validated. RPS6KA6, RSPO3, and TMEM132E are significantly overexpressed in HCC tissues (Fig. 5). Images of immunohistochemically stained tissue samples showed that RPS6KA6 expressed in normal tissues with medium staining but that it was significantly overexpressed in HCC tumor tissues with high staining.

## 4. Discussion

Currently, growing data suggested that ncRNAs, particularly lncRNAs and circRNAs play a significant role in the development of numerous diseases in various ways. RNA molecule with a length of >200 nucleotides that does not have an open reading frame and encodes a protein is referred to as a lncRNA.^[16]^ circRNA is an endogenous ncRNA, usually produced by reverse splicing of RNA and cyclization of exons or introns. It has a covalently closed-loop structure. In eukaryotes, circRNA is relatively prevalent and has a highly conservative evolutionary history.^[17]^ Both lncRNA and circRNA can isolate miRNA by interacting with miRNA, inhibit the interaction between miRNA and target mRNA, and promote ceRNA activity. lncRNA, circRNA, and mRNA are effectively cross-talking with miRNA in the ceRNA mechanism, and they are controlled in a variety of pathophysiological processes in both plants and animals.

In this study, we established a ceRNA composed of lncRNA, circRNA, miRNA, and mRNA. This network was composed of 6 lncRNAs, 13 circRNAs, 10 miRNAs, and 55 mRNAs. Among 55 mRNA, CPEB3, EFNB3, FATA4, GHR, GSTZ1, KLF8, MFAP4, PAIP2B, PHACTR3, PITPNM3, RPS6KA6, RSPO3, SLITRK6, SMOC1, STEAP4, SYT1, TMEM132E, TSPAN11, and ZFPM2 were intimately bound up with OS in HCC patients. CPEB3, GHR, GSTZ1, and KLF8 were especially crucial molecules in the onset and progression of diseases and have been widely investigated in HCC or other forms of malignant tumors.

CPEB3 and its upstream ceRNA network hsa_circ_0050898 (circRNA ACTN4)/MIR503HG-miR-195-5p-CPEB3 was important in multiple diseases. CPEB3 is a sequence-specific RNA-binding protein that binds to polyadenylation elements in the cytoplasm. Generous studies have proved that CPEB3 was essential for the development of HCC.^[18]^ Besides, miR-18a-5p, miR-9-5p, miR-20b-5p, miR-224, miR-452-3p, and miR-107 promoted HCC cell proliferation, migration, and invasion by targeting CPEB3.^[19–25]^ In colorectal cancer, CPEB3 knockout in CRC cells enhances CD163 + TAM polarization and M2-like TAM-derived cytokine secretion. interleukin-6 of TAMs and CCL2 of CRC cells were measured in the Luminex test to evaluate the secretory signal between stably transfected CRC cells and THP-1 macrophages.^[26]^ In Fang et al’s study, in the reproductive system, especially in the oocytes of primary and secondary follicles, CPEB3 is highly expressed. Although there was no significant difference in the total weight of these mice, there were significant differences in the morphology of wild-type and CPEB3 mutant ovaries and the average weight and size of CPEB3 mutant ovaries compared with wild-type ovaries. So, due to its regulation of Gdf9 expression in oocytes, CPEB3 is crucial for the growth of ovarian follicles and female fertility.^[27]^ hsa_circ_0050898, which was derived from the ACTN4 gene’s exons 1 to exons 20, was overexpressed in intrahepatic cholangiocarcinoma. circRNA ACTN4 upregulates the expression of Yes-related protein 1 by absorbing miR-424-5p. High circACTN4 expression was linked to inferior outcomes after intrahepatic cholangiocarcinoma resection and increased tumor growth and metastasis both in vitro and in vivo.^[28]^

GHR and its upstream ceRNA network MIR99AHG/hsa_circ_0050898/MIR503HG-hsa-let-7b-5p/hsa-let-7c-5p/hsa-miR-195-5p/hsa-miR-424-5p-GHR have been widely investigated in various diseases. GHR is a typical class I cytokine receptor. In children and teenagers, GHR regulates development, metabolism, and aging in a significant physiological way.^[29]^ Gao et al found that GHR knockdown enhances the sensitivity of HCC cells to sorafenib, and the inactivation of PI3K/AKT/ERK1/2 signaling pathway may be its potential mechanism.^[30]^ Haque et al findings implied that although some animals with the LiGhr-/- genotype developed HCC, these tumors were noticeably less numerous than those that appeared in mice with intact GHR expression in the liver, strongly indicating that GHR expression in the liver may increase HCC tumor burden.^[31]^ In Kaseb et al research, 49.5% of patients with HCC had considerably high GH levels, and these individuals had more aggressive diseases and worse clinical outcomes (*P* < .0001). In vitro, siRNA or pegvisomant used to block GHR signaling resulted in significant inhibitory cellular effects.^[32]^

In conclusion, this work offered a preliminary overview of the DElncRNA, circRNA, and mRNA in HCC. This study gives clinical therapy alternatives and advances our understanding of the pathophysiology of HCC. The precise role of lncRNA and circRNA in HCC, however, has to be confirmed by in vivo and in vitro research.

## Author contributions

**Conceptualization:** Fuyin Zhou.

**Investigation:** Qingsong Kang.

**Methodology:** Junbo Ma, Jie Cai, Ying Chen.

**Supervision:** Feibo Li.

**Visualization:** Kai Qu.

**Writing – original draft:** Fuyin Zhou.

**Writing – review & editing:** Feibo Li.
